# Abdominal hernia mesh repair in patients with inflammatory bowel disease: A systematic review

**DOI:** 10.1007/s00423-022-02638-x

**Published:** 2022-08-10

**Authors:** Michael El Boghdady, Béatrice Marianne Ewalds-Kvist, Aggelos Laliotis

**Affiliations:** 1 Department of General Surgery, Kingston Hospital, London, UK; 2 University of Edinburgh, Scotland, UK; 3 Stockholm University, Stockholm, Sweden; 4 University of Turku, Turku, Finland; 5 Department of General Surgery, Croydon University Hospital, London, UK

**Keywords:** Inflammatory bowel disease, Crohn’s disease, Ulcerative colitis, Hernia repair, Surgical mesh

## Abstract

**Background:**

Postoperative hernia-repair complications are frequent in patients with inflammatory bowel disease (IBD). This fact challenges surgeons’ decision about hernia mesh management in these patients. Therefore, we systematically reviewed the hernia mesh repair in IBD patients with emphasis on risk factors for postoperative complications.

**Method:**

A systematic review was done in compliance with the PRISMA guidelines. A search was carried out on PubMed and ScienceDirect databases. English language articles published from inception to October 2021 were included in this study. MERSQI scores were applied along with evidence grades in agreement with GRADE’s recommendations. The research protocol was registered with PROSPERO (CRD42021247185).

**Results:**

The present systematic search resulted in 11,243 citations with a final inclusion of 10 citations. One paper reached high and 4 moderate quality. Patients with IBD exhibit about 27% recurrence after hernia repair. Risk factors for overall abdominal septic morbidity in Crohn’s disease comprised enteroprosthetic fistula, mesh withdrawals, surgery duration, malnutrition biological mesh, and gastrointestinal concomitant procedure.

**Conclusion:**

Patients with IBD were subject, more so than controls to postoperative complications and hernia recurrence. The use of a diversity of mesh types, a variety of position techniques, and several surgical choices in the citations left room for less explicit and more implicit inferences as regards best surgical option for hernia repair in patients with IBD.

## Background

Altogether, 2.5 million residents in Europe and 1 million in the USA are projected to have inflammatory bowel disease (IBD) in a near future; IBD is on the rise also in Asia, South America, and Middle East constituting a global burden [1]. IBD comprises Crohn’s disease (CD) with an incidence of 3 to 20 cases per 100,000 [2, 3] as well as ulcerative colitis (UC) with an incidence of 9 to 20 cases per 100,000 persons per year [4]. Pre-existing IBD predicts further hernia surgery [5].

An abdominal wall hernia is a weakness in the muscles of the abdominal wall through which a portion of organ or tissue can protrude and an incisional hernia (IH) after abdominal surgery is a frequent complication following laparotomy. Surgical repair of hernia is recommended for circumvention of complications and symptoms. This is the only absolute treatment, which can be done through an open or laparoscopic approach and with possible use of mesh prothesis. Abdominal and IH repair with primary suturing have a higher recurrence rate than mesh repair [6]. Yet, the use of mesh as a foreign body can lead to complications in forms of pain, infection, fistula, bowel injury, and bowel adhesions [7]. So far, newer models of mesh products have evolved over time, and an increased attention is directed towards their manufacturer for avoidance of product-related adverse complications after hernia repair.

Furthermore, patients with IBD are at risk for intestinal difficulties like obstruction, bowel perforation, fistula, toxic megacolon, and infective flares [8]. As the risk of postoperative hernia-repair complications is high, the surgeon’s decision for mesh management for patients with IBD constitutes a factual challenge in clinical practice. We aimed to systematically review the outcomes of hernia repairs in patients with IBD. We concentrated on correlations of risk factors and postoperative complications with hernia recurrence.

## Methods

### Protocol

The research protocol was registered with PROSPERO register for systematic reviews (CRD42021247185). A systematic review was performed in compliance with the PRISMA (preferred reporting items for systematic review and meta-analysis) guidelines [9] along with GRADE recommendations [10, 11].

### Search strategy

**Fig. 1:**
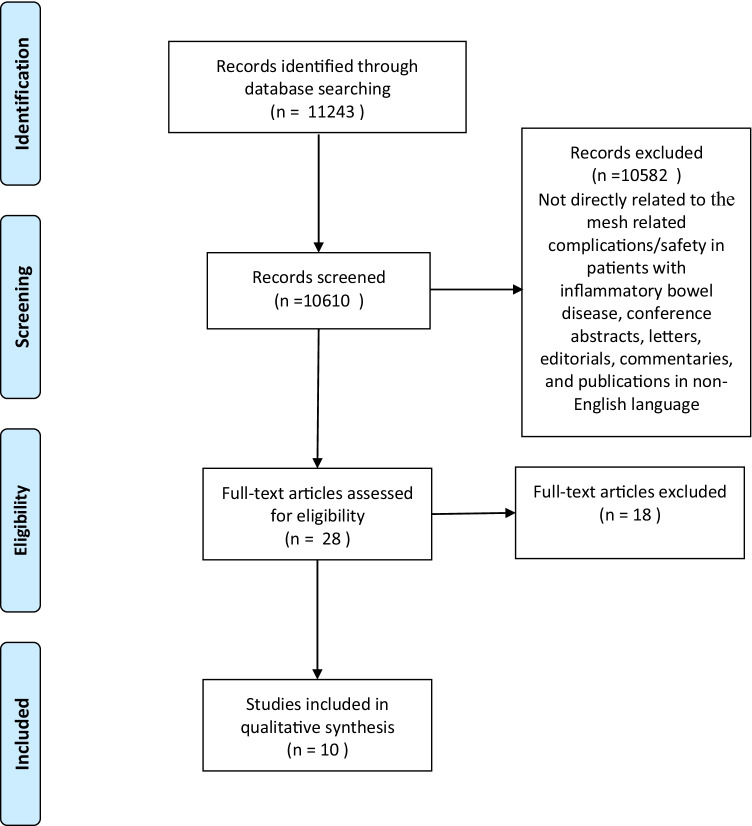
Flow diagram of the systematic search

A literature search was carried out on PubMed and ScienceDirect for articles published from inception to October 2021 (Fig. 1). Search terms used were chosen from the list of MeSH (Medical Subject Headings). The search algorithm used were mesh term for Crohn’s disease and surgical mesh, ulcerative colitis and surgical mesh, inflammatory bowel disease and surgical mesh, Crohn’s disease and hernia, ulcerative colitis and hernia, and inflammatory bowel disease and hernia.

### Inclusion and exclusion criteria

Citations directly related to abdominal hernia repair with mesh in patients with inflammatory bowel disease were included in this study.

Studies that did not clearly provided information about the mesh related complications/safety in patients with inflammatory bowel disease were excluded. Conference abstracts, letters, editorials, commentaries, protocols, experimental animal trials, and non-English publications were excluded.

### Quality assessment

The retrieved citations were read in full text for further assessment for eligibility. Quality assessments and quality of studies were applied using The Medical Education Research Study Quality Instrument (MERSQI) [12] which contains 10 items that reflect 6 domains of study quality including study design, sampling, type of data, validity, level of data analysis, and outcomes. For the assessment of the validity of evaluation instrument, we focused on face validity, limitations, and correlations with other instruments. The MERSQI score represents the mean of two independent assessors’ quality estimations of each citation. MERSQI produces a maximum score of 18 with a potential range from 5 to 18. The maximum score for each domain was 3. The mean quality score was calculated to be 13.83 (*SD* = 1.46) = moderate quality score of citation ~ 0.14. High-quality score was M + 1 SD ~ 15.5 and low-quality score was M-1 SD ~ 12.5. Very low quality was M-2 SD ~ 11.

### Evidence grading

Quality of evidence for grading the studies was based on the principles elaborated by GRADE. Consequently, the evidence grading was based on criteria for using GRADE, comprising four grades:

- Evidence grade I: strong scientific evidence based on at least 2 studies with high evidential value or a systematic review/meta-analysis with high evidential value
- Evidence grade II: moderate scientific basis: a study with high evidential value and at least 2 studies with moderate evidential value
- Evidence grade III: low scientific evidence: a study with high evidential value or at least 2 studies with moderate evidence value
- Evidence grade IV: insufficient scientific evidence: 1 study with moderate evidence and/or at least 2 studies with low evidential value

### Risk of bias within and across studies

We decreased the risk of bias by assessing quality in a blind manner by two authors, independently. If the assessment scores did not agree, we calculated the mean of the given scores. The calculated interrater reliability was significant (*p* < 0.001). We controlled for accumulated risk of bias by calculating and grading the body of evidence of the findings by determining the limits of the four grades by taking the sample’s mean score M as we maintain a moderate confidence about the result’s effect (II). Then we determined M ± 1 SD for a higher level of confidence in the effect (I) as oppose to taking M-1 SD for a lower level of confidence in the effect (III) and finally M-2SD indicated a very low confidence in the effect (IV) (Cf 12). The effect refers to the best result of the use of a certain type of technique for repair of hernia in patients with IBD. The risk of bias was likewise reduced by exclusion of citations with evidence grades III and IV in the grading, i.e., only citations of high (I) and moderate (II) quality were included in the final result.

## Results

### Citation selection and characteristics

The present systematic search resulted in 11,243 citations, out of which relevant citations were extracted after scanning their titles and abstracts (Fig. 1). The inclusion and exclusion criteria were applied and duplicated citations were excluded. A final 10 citations were suitable relative to the research rational and the articles’ full texts were read for further evaluation. The mean number of years was 11.1 years (SD 10.71 years), ranging from 1 to 38 years. The interrater reliability for quality assessment was *r*_*s*_ = 0.94; *p* < 0.001. The tabular analysis of the citations for patients with IBD is presented in Table 1 which comprises details about studies, journals, quality scores and evidence grades of the studies. Furthermore, the citations’ aims, kind of hernia, hernia-repair technique, type of mesh, findings, and complications are described [13–22].

**Table 1:** The tabular analysis of the citations for patients with IBD

Author (year) and journal	Study type, patients and data years^1^	Objective	Technique	Type of mesh	Findings/complications	Follow-up	Q S/E G^2^
Sugerman et al. (1996) THE AMERICAN JOURNAL OF SURGERY	Cohort study (98 patients/18 UC) Data covers the years 1982 to 1993	Evaluation of IH prefascial/onlay polypropylene mesh repair post RYGBP vs total abdominal colectomy and IPAA for UC	Primary closure + prefascial/onlay mesh repair	Polypropylene	Complications occurred in 35% of the patients such as haematoma, seroma, minor wound infections. Major wound infections 5%. No enterocutaenous fistula or chronic wound drainage reported. Recurrence 4%. No reported difference between groups	Mean 20 + 2 months (range 6 to 104)	13.5; III
Aycock et al. (2007) JOURNAL OF WOUND, OSTOMY & CONTINENCE NURSING	Series (11 patients, 9 had IBD) From 2004 to 2006	The use of Acellular dermal matrix in parastomal hernia repairs	Onlay repair (72%) and Inlay techniques	Acellular dermal matrix	Complications: 2 (18%) wound infections Recurrence: 2 (18%) clinical recurrence. 1 subclinical/CT proven recurrence	Mean 8.7 months (range 1 to 21 months)	11; IV
Taman et al. (2009) DISEASES OF COLON & RECTUM	Cohort study, 13 IBD patients with parastomal hernia From 2006 to 2007	To study patients with parastomal hernia, repair of stoma site and abdominal wall with hADM	Neofascia creation using two separate layers of human acellular dermal matrix reconstructed with human acellular dermal matrix	human Acellular Dermal Matrix: hADM (LifeCell, Branchburg, NJ)	Seroma formation, incisional separation (2 patients each, 15.4 percent), and superficial wound infection (1 patient, 7.7 percent). There were two cases of asymptomatic hernia recurrence as determined by CT	M 290 (range 137–509) days	14; II
Maman et al. (2012) ANNALS OF PLASTIC SURGERY	Cohort study A total of 59 patients, 38 (64.4%) had IBD From 1999 to 2007	To study the modified Rives-Stoppa repair for complex incisional hernias	Rives-Stoppa repair. Mesh is anchored in the retrorectus position/sublay via nonabsorbable sutures	ePTFE and poly-propylene	Complications: seroma (6.8%), wound/ mesh infection (6.8%) Recurrence: (1.7%) None of the patients developed bowel fistula	Mean 40.0 mo. (range, 6.7–117.6 mo.)	12.75; III
Wang et al. (2016) THE AMERICAN SURGEON	Cohort study (38 patients) all had IBD From 2007 to 2013	The study of the ventral hernia repair with retrorectus mesh reinforcement in patients with IBD	Sublay mesh repair, retrorectus mesh rein-forcement with mid-line reapproximation of fascia with restoration of linea alba (LA). Rives-Stoppa retrorectus repair if hernia size did not preclude repair of LA without a component parting and adequate mesh overlap in confines of the rectus sheath	Altogether 22 (58%) biologic mesh and 16 (42%) synthetic mesh	Altogether 3 wound infections and 1 seroma/hematoma. Surgical site infection occurred in 7 (18.4%) patients. No reported mesh infection Recurrence: 3 (9.4%) No instances of postoperative intestinal complications or enterocutaneous fistulae	3–4 weeks, 3 mo., 6 mo. 1 year and then annually M follow-up (FU) 32 mo. (3–83 mo.) + remaining 32 cases with M FU of 37 mo (range, 13–83 mo.)	13.5; III
Heimann et al. (2017) THE AMERICAN JOURNAL OF SURGERY	Cohort study (170 patients) all had IBD From 1976 to 2014	To determine the outcome of incisional hernia (IH) repair in patients with IBD and the factors that correlate with recurrence of IH	Open repair (92.4%), laparoscopic (7.6%). Primary suture repair (38.2%)	Biologic mesh (7.6%), synthetic mesh (50.6%), biologic and synthetic mesh (3.5%), onlay mesh repair (59%) and sublay (41%)	Altogether 61 had onlay, 1 got mesh infection; 31 patients had inlay synthetic mesh repair. In 3 cases, late onset enterocutaneous fistulas were identified after IH repair with synthetic mesh inlay. Hernia recurrence in 46 cases; 38 of these patients underwent a second IH repair and 10 (26%) recurred again	Mean 56 months	15: II
Beyer-Berjot et al. (2020) WORLD JOURNAL OF SUGERY	Retrospective multicentre controlled study (234 with 114 Crohn’s disease patients) From 2000 to 2017	To assess the risk of septic morbidity (SM) after ventral hernia mesh repair in patients with Crohn’s disease (CD)	All types of VH repair were used, provided that they included mesh positioning. All types of mesh were used (absorbable, perma-nent synthetic or biological) with all means of mesh fixation (threads or tackers). Mesh was placed as IPOM or sublay (i.e., retro-rectus) by laparosco pic or open approach	Permanent synthetic mesh in 95 patients with CD vs. 109 controls. Absorbable mesh in 6 CD patients vs. 7 controls and biological mesh in 11 CD patients vs. 4 controls	Altogether 12 patients (10.4%) had chronic mesh infection, including 8 intestinal fistulas involving the mesh (7%), leading to late reoperations in 9 cases (7.8%) and mesh withdrawal in 6 cases (5.3%). SM occurred in 21 CD patients (18.4%); 11 patients (9.6%) experienced short-term abdominal SM with either wound (7%) or intra-abdominal sepsis (2.6%), leading to two reoperations (1.7%) and one CT-guided drainage (0.9%). Recurrence: 16 (14%) in patients with CD	Median follow-up of 21.3 months (1–132)	16.25; I
Heise et al. (2021) BMC SURGERY	Retrospective analysis ( total of 223 patients, 34 had IBD) From 2005 to 2018	To study the role of IBD as perioperative risk factor in open ventral hernia repair (OVHR) and the role of IBD on hernia recurrence	IH repair was performed as OVHR with mesh augmentation in sublay position	A PVDF-mesh (DynaMesh®, FEG-Textiltechnik) was placed in sublay position on peritoneum and posterior rectus sheath	OVHR in patients with IBD carried higher rate of intraoperative blood transfusions, major complications, and postoperative relaparotomies. IBD predicts per se major postoperative morbidity. Hernia recurred in 9 out of 34 patients: 15 suffered from UC and 19 from CD. UC was often associated with IH recurrence compared to CD	Median 36 months	14.5; II
Horesh et al. (2021) EUROPEAN JOURNAL OF GASTRO-ENTEROLOGY & HEPATO-LOGY	Retrospective analysis of a pro-spective database (5467 IBD cases, 26 got inguinal hernia repair) From 2008 to 2019	To assess surgical outcome in patients with IBD with inguinal hernia repair and to assess risk factors	Prolene mesh was used to reconstruct the inguinal canal and close the hernia site defect	Prolene mesh	Three intraoperative complications were recorded (1 bladder injury and 2 orchiectomies). Postoperative complications occurred in eight patients most commonly wound related (three wound infections and one postoperative seroma). One patient required reoperation due to bowel obstruction. Hernia recurrence was seen in two patients during follow-up	Follow-up time mean 2.55 years	14: II
Perl-Mutter et al. (2021) HERNIA	Cohort retrospective study of 40 patients with CD From 2014 to 2018	To describe the post-operative results and healthcare resource utilization after incisional hernia repair with synthetic mesh in patients with CD	Open incisional hernia repair with extra-peritoneal synthetic mesh	38 had synthetic mesh placed in sub-lay position, 2 had onlay, 36, had medium weight polypropylene mesh; 39 got repair with 1 mesh to cover all defects,1 had a medium weight mesh to repair the para-stomal defect, a heavy weight mesh was used to repair the midline defect	A total of 16 patients had complications or recurrence. 6 were readmitted in 30 days, 4 had abdominal pain, nausea, and vomiting, 1 diarrhea and 1 SSI. 1 had revision of hernia repair 8 days postoperatively for small bowel obstruction using coated medium weight polypropylene mesh, and got 35 days after enterocutaneous fistula with mesh excision; 3 got SSI in 30 days, 1 superficial and 2 deep. 4 had SSO: 1 got a small area of fat necrosis, 1 got a short incisional skin separation, 1 had seroma, and 1 wound cellulitis. During follow-up of 42 mo., 8 patients had recurrence of hernia, at 18 mo. 2 of these 8 patients had repair	Follow-up Md 42 mo	13.25; III

^1^Data in studies covering mean 11.1 years (*SD* = 10,71 years). ^2^*Q S/E G*, quality scores and evidence grade; *I High quality*, = 13,83 + 1,46 = 15,29 = 15,5; *II Moderate quality*, = 13,83 = 14; *III Low quality*, = 12,37 = 12,5; *IV Very low quality*, = M-2SD = 10,91 = 11; *Mann–Whitney U*, 0 < 2 between I + II and III + IV = *p* < .05. Abbreviations: *RYGBP= Roux-en-Y* gastric by pass, *IPAA=* ileal pouch anal anastomosis, *hADM* =human acellular dermal matrix, *IPOM* =intraperitoneal onlay, *OVHR* =open ventral hernia repair, *IH=* incisional hernia; *SM* =septic morbidity, *SSI=* surgical site infection, *SSO=* surgical site occurences

### Results of quality and evidence-grade assessments

Out of 10 citations, one reached high quality (grade I), 4 moderate quality (grade II), 4 low quality (grade III), and 1 very low quality (grade IV). Papers with evidence grades I and II were considered for evidence-based outcome. The evidence grades were determined as follows: I high quality = 13.83 + 1.46 = 15.29 = 15.5; II moderate quality = 13.83 = 14; III low quality = 12.37 = 12.5; IV very low quality = M-2SD = 10.91 = 11. The difference between I and II and III and IV was significant (*p* < 0.05) (Table 1).

### Results of individual studies

Beyer-Berjot et al. [13] assessed the risk of septic morbidity (SM) in patients with CD after mesh repair for ventral hernia (VH). The study was a 1:1 matched case–control analysis and elective mesh repair for VH was performed. Controls were non-IBD. All kinds of VH repair involving mesh positioning were included. Absorbable, permanent synthetic or biological mesh and thread or tacker mesh fixation were involved. The mesh was positioned as intraperitoneal onlay (IPOM) or sublay in a laparoscopic or open approach. No heavy weight mesh was used. Only type I with pores larger than 75 microns were employed, whether with polypropylene, composite polypropylene and ePTFE, and composite polypropylene and hydrogel or polyester.

Abdominal septic morbidity (ASM) connected to hernia repair, indicated inflamed skin, acute leaking, fistula or abscess in subcutaneous or peri-prosthetic space and fever (38.5 °C) with no other causes. ASM occurred in 21 out of 114 CD patients; 11 patients experienced short-term ASM with wound (7%) or intra-abdominal sepsis (2.6%) with two reoperations and one CT-guided drainage. After follow-up, 12 patients experienced chronic mesh infection, including 8 intestinal fistulas with mesh involvement and late reoperations in 9 cases and mesh withdrawal in 6 cases. Fourteen patients underwent reoperation for CD recurrence. Risk factors for ASM in CD patients were malnutrition, midline incision site of hernia, biological mesh, and digestive concomitant procedure. The B3 phenotype, anti-TNF therapy, and corticosteroids were not associated with a higher risk of postoperative sepsis.

The mesh was permanent synthetic in 95 CD patients vs. 109 controls, absorbable in 6 CD patients vs. 7 controls, and biological in 11 CD patients vs. 4 controls. Short-term severe postoperative morbidity was similar in CD and control groups but CD patients were at higher susceptibility of abdominal SM, both short-term and long-term as well as at risk of entero-prosthetic fistula and mesh withdrawals, more so than controls. Hernia recurrence was similar in both groups. No patient died but CD is a risk factor for SM after mesh repair in VH.

Heimann et al. [14] studied 1000 patients with IBD undergoing open bowel resection. Of these, 203 developed IH and outcomes of 170 patients with IBD, who underwent IH repair, are reported in the study; 92 suffered from UC and 78 patients endured CD. The use of mesh, its placement, and incidence of post-operative complications were similar in both groups. Patients with CD had higher rate of bowel resection and/or presence of ileostomy during hernia repair.

Sixty-one patients had IH repair with onlay synthetic mesh. One patient underwent mesh infection, removal of mesh and complex abdominal wall reconstruction. Thirty-one patients had inlay synthetic mesh repair and 1 UC and 2 CD developed late-onset enterocutaneous fistula 3–7 years postoperatively requiring reoperation, bowel resection, and removal of mesh. Hernia recurrence after IH repair was found in 46 cases; 38 patients underwent a second IH repair out of whom 10 recurred again and needed further surgery. Patients with UC undergoing primary repair had a higher recurrence rate than those enduring mesh repair. Patients with CD had similar recurrence rates for primary IH repair while those undergoing mesh repair had a higher rate of recurrence than patients with UC.

It was found that number of previous bowel resections, primary repair, use of biological mesh for reconstruction, postoperative complications, septic complications, and postoperative wound infection correlated with a higher recurrence of hernia after IH repair. Yet, the only significant independent predictor by means of multivariate statistics for recurrence of hernia after IH repair was the number of previous bowel resections.

After IH repair, about 27% of patients relapsed. IBD patients with second repair also had a recurrence rate of 26%. Similar rates have been reported for non-IBD patients. In sum, the number of previous bowel resections, primary repair, use of biological mesh, postoperative complications, septic complications, and postoperative wound infection correlated with recurrence of hernia after IH repair. Multiple bowel resections lead to recurrent IH. The use of synthetic mesh for IH repair in UC decreased recurrence rate. In patients with CD, synthetic mesh did not improve the recurrence rate over primary repair. Inlay synthetic mesh for IH repairs in patients with IBD has a potentially higher risk for late-onset enterocutaneous fistula.

Heise et al. [15] disclosed that patients with IBD have a high life-time risk for abdominal surgery and incisional hernias (IH). The postoperative course was studied of non-IBD (*n* = 199) vs. IBD (*n* = 34) patients with IH repair: 15 patients presented UC and 19 presented CD. The IH repair consisted of open ventral hernia repair (OVHR) with mesh augmentation in sublay position in form of PVDF on peritoneum and posterior rectus sheath.

The perioperative data revealed in IBD group compared to controls, higher rates of intraoperative blood transfusions, major complications, and postoperative relaparotomies.

During follow-up, hernia recurrence occurred in 9 IBD patients (almost 27%). An association of UC, history of more than 1 bowel resection, and extraintestinal manifestation with occurrence of recurrent hernia were found. UC was recognized as associated with IH recurrence, more so than CD patients. Patients with IBD showed higher rates of major complications after OVHR, but incidence of overall complications was not elevated compared to those non-IBD patients. By means of multivariate binary regression, the presence of IBD (*HR* = 4.19, *p* = 0.007) was the single independent predictor of major postoperative morbidity (Tables 1 and 2).

**Table 2:** Summary of risk factors and complications relative to hernia repair in patients with IBD

Study	Patients with CD	Patients with UC	Patients with IBD	Controls	Risk factors for patients with IBD of post-operative complications	Statistics p <	Hazard ratio (HR)
Beyer-Berjot et al	114		114	120	• CD > UC for septic morbidity • Entero-prosthetic fistula • Mesh withdrawals • Biological mesh • Malnutrition • Concomitant procedure • Overall abdominal septic morbidity (SM) • Short-term abdominal SM • Long-term abdominal SM • Hernia recurrence in CD patients 14%	.001 .01 .011 .0001 .004 .004 .001 .025 .002	
Heimann et al	78	92	170		• *n* of bowel resections prior to hernia repair predicted recurrence of IH • Biologic mesh • Recurrence 27%	.01 .01	*HR* = 1.59
Heise et al	19	15	34	199	• Patients with UC suffer more from hernia recurrence than those with CD • More than 1 bowel resection plus extraintestinal manifestations with hernia • Intraoperative blood transfusion • Major complications • Postoperative relapatomies • Intensive care due to post-operative complications • Intensive care morbidity predictor • Recurrence 26.5%	.02 .02 .001 .001 .006 .001 .001	*HR* = 11.7 > *HR* = 1.0 *HR* = 11.68 *HR* = 13.31 *HR* = 3.5 *HR* = 3.67
Horesh et al	14	12	26	76	• Surgery duration risk factor for IBD patients • Patients with IBD more postoperative complications than controls	.0001 .03	

^1^Cox proportional hazard regression: *HR* hazard ratio

Horesh et al. [16] studied 26 out of 5467 IBD patients in their institution; 14 suffered from CD and 12 patients from UC. This cohort endured IH repair and was matched to 76 controls who also experienced IH. Patients with CD had larger hernia defects (> 5 cm) than those with UC.

Prolene mesh was employed to reconstruct the inguinal canal and to close the hernia site defect. There was no significant difference between number of patients with CD and UC who underwent laparoscopic or open surgery.

Postoperative complications followed in 8 patients: three wound infections and one postoperative seroma. One patient needed reoperation due to bowel obstruction. Hernia recurrence happened in two patients during follow-up. Postoperative complication rates were higher in IBD patients compared to those non-IBD undergoing IH repair. However, open IH repair showed similar recurrence rates when compared to laparoscopic repair. Surgery duration correlated significantly with postoperative-morbidity risk. Gastroenterologists’ and surgeons’ awareness of increased risk for surgical complications in patients with IBD patients is required.

### Synthesis of results

The summery of risks and post-surgery complications in patients undergoing hernia repair as well as significant differences in results between patients with IBD and their controls is presented in Table 2. In general, ~ 27% of patients with IBD were subject to hernia recurrence after hernia repair had a mean of 36 (range 36–56) months of follow-up time.

## Discussion

We systematically reviewed outcomes of hernia repairs in patients with IBD with emphasis on consequences for postoperative complications. After assessing citations with high and moderate quality, four citations formed in combination a base for moderate evidence for our results. We focused on findings based on univariate and multivariate significant factors leading to recurrent hernia repair and post-surgery complications in patients with IBD. In these patients, corticosteroids and anti-TNF agents have been associated with increased overall postoperative infection risk as well as intra-abdominal infection [23]. In addition, mesh contact with an inflamed bowel in hernia repair can cause complications such as adhesions, intestinal obstructions, and enterocutaneous fistulae.

Different types of mesh were used in our study. Two out of four citations considered biologic mesh a risk factor for complications in post-hernia repair. However, Beyer-Berjot et al. used absorbable, permanent synthetic, or biologic mesh with fixation either by threads or tackers [13]. Only type I with pores larger than 75 microns was employed, whether with polypropylene, composite polypropylene, and ePTFE as well as in composite polypropylene and hydrogel or polyester. The researchers concluded that biologic mesh should be avoided. Heimann et al. used biologic and synthetic mesh and onlay as well as sublay mesh repair were applied [14]. Yet, biologic mesh was found to be a risk factor for complications. Our finding agreed with those of a previous study that claimed that while biologic mesh is derived from decellularized human, bovine, and porcine tissue, it constitutes in its final form a collagen matrix, which impacts biocompatibility, foreign body response, and immunogenic potential of the graft [24]. Researchers also found that biologic and biosynthetic mesh should not be used in a bridging situation [25] and did not reveal any explicit advantages of biologic and biosynthetic meshes in inguinal hernia repair. Furthermore, no evidence was revealed for the use of biologic or biosynthetic meshes in the prevention of incisional and parastomal hernias.

The technique of mesh placement has continuously been debatable, based on the patient’s condition and surgeon’s preference. Beyer-Berjot et al.’s mesh was positioned as IPOM or sublay [13]. Heise et al. used polyvinylidene fluoride PVDF-mesh which was placed in sublay position on peritoneum and posterior rectus sheath [15]. This is a textile-based German mesh with a hernia recurrence was 26.5% in patients with IBD. Horesh et al. used prolene (polypropylene) mesh, which is the most common type of synthetic hernia mesh [16]. It is made from plastics and may reduce the chances of a hernia recurrence. It has previously been evidenced that permanent synthetic mesh when placed in an extraperitoneal position is safe for VHR in a contaminated field along with conferring a significantly lower rate of surgical site infection and recurrence compared to those biologics or bioabsorbable meshes [26]. In complex abdominal wall hernia repair with incarcerated hernia, parastomal hernia, infected mesh, open abdomen, entero-cutaneous fistula, and component separation technique, it has been indicated that biologic and biosynthetic meshes were not superior to synthetic meshes [25]. It is advisable to avoid placement of mesh in direct contact with the bowel, especially in patients with IBD.

Different types of hernia-repair techniques used for patients with IBD undergoing such repair also varied from citation to citation. As regard surgical technique for patients with IBD, a previous study claimed that with growing expertise in laparoscopic surgery, the minimally invasive approach is at least comparable to the open access surgery as regards long-term outcome in patients with CD [27]. Heimann et al.’s patients were subject to a laparoscopic or open approach with no difference between those with CD and UC. Heise et al.’s patients with IBD were all subject to an IH performed as an OVHR. Out of Horesh et al.’s patients with IBD, 61.5% were subject to an open approach of inguinal hernia repair. In other words, both laparoscopic and open approaches were applied for hernia repair. The current trends in laparoscopic surgery for UC were previously reviewed [28] and it was found that, although laparoscopic surgery sometimes requires a longer operation, it provides better short-term benefits compared to open surgery comprising shorter hospital stays and fasting times, as well as better cosmesis. The long-term benefits of laparoscopy include better fecundity in young females. Some surgeons favor laparoscopic surgery even for severe acute colitis due to fewer postoperative complications compared to open.

One of Beyer-Berjot’s risk factors for post-surgery complications in form of septic morbidity was malnutrition in accordance with results from a previous research that showed that poor nutrition significantly increased the risk of infectious complications such as anastomotic leak, intra-abdominal abscess, enterocutaneous fistula, or wound infection in patients with IBD [29]. Heimann et al. indicated that number of bowel resections prior to hernia repair predicted recurrence of IH [14]. It has also been found that the incidence of IH was 21% for patients with UC and 20% for patients with CD. Statistically significant risk factors for development of IH were among others, wound infection, and a history of previous bowel resection. Hernia recurrence did not differ between an open vs. laparoscopic approach in patients with IBD [30]. However, hernia recurrence is a time-dependent process [31]; Heise et al. found that IBD patients displayed a hernia recurrence rate of about 27% during a follow-up of 36 months. Heimann et al. did their follow-up during 56 months also with 27% hernia recurrence. Furthermore, IBD stands as a significant risk per se for major postoperative morbidity after OVHR. In addition, individuals with IBD show high rates of hernia recurrence over time with UC patients being more prone to recurrence than patients with CD. Horesh et al.’s postoperative complications in patients with IBD were 30.7% vs 11.8% in controls. Yet, only 2 out of 26 patients with IBD had hernia recurrence.

The study is limited by the fact that only a few citations were available in our final selection and their retrospective long-term data sampling (e.g., 38 years) nature did not reach high quality and evidence grade 1. During such a long time, a substantial mesh development takes place and continuous improvement in material and techniques are expected to better fit the hernia-repair needs for patients with IBD. 

Mesh-defect-area-ratio, fixation techniques, tissue elasticity, and the hernia size under pressure can be subject for future studies for the repair of large, recurrent, and complex incisional hernia in IBD patients [32]. In addition to the possible use of tools for risk stratification, e.g., using the CEDAR app [33]. There is also claimed to be a difference in hernia-repair recurrence between patients with CD and UC, a subject that needs more clarification in future research.

## Conclusion

Patients with IBD were subject, more so than controls, to postoperative complications and hernia recurrence. The use of a diversity of mesh types, a variety of position techniques, and several surgical choices in the citations left room for less explicit and more implicit interpretations as regards best surgical option for hernia repair in patients with IBD.
