# Correction to: Abdominal hernia mesh repair in patients with inflammatory bowel disease: A systematic review

**DOI:** 10.1007/s00423-022-02699-y

**Published:** 2022-10-03

**Authors:** Michael El Boghdady, Béatrice Marianne Ewalds‑Kvist, Aggelos Laliotis

**Affiliations:** 1 Department of General Surgery, Kingston Hospital, London, UK; 2 University of Edinburgh, Scotland, UK; 3 Stockholm University, Stockholm, Sweden; 4 University of Turku, Turku, Finland; 5 Department of General Surgery, Croydon University Hospital, London, UK

**Correction to** **:** **Langenbeck's Archives of Surgery**

https://doi.org/10.1007/s00423-022-02638-x

The original version of this article unfortunately contained a small mistake on page 11, the in-text citation [17] should be changed to [14].

The corrected in-text citation is shown below:

Heimann et al. indicated that number of bowel resections prior to hernia repair predicted recurrence of IH [14].

The original article has been corrected.

